# Dynamic Changes in Arabinogalactan-Protein, Pectin, Xyloglucan and Xylan Composition of the Cell Wall During Microspore Embryogenesis in *Brassica napus*

**DOI:** 10.3389/fpls.2019.00332

**Published:** 2019-03-28

**Authors:** Patricia Corral-Martínez, Azeddine Driouich, Jose M. Seguí-Simarro

**Affiliations:** ^1^COMAV – Universitat Politècnica de València, Ciudad Politécnica de la Innovación, Valencia, Spain; ^2^Laboratoire Glycobiologie et Matrice Extracellulaire Végétale, UPRES-EA 4358, Fédération de Recherche Normandie-Végétal – FED 4277, Université de Rouen Normandie, Mont-Saint-Aignan, France

**Keywords:** androgenesis, cell wall, doubled haploid, electron microscopy, immunogold labeling, rapeseed

## Abstract

Microspore embryogenesis is a manifestation of plant cell totipotency whereby new cell walls are formed as a consequence of the embryogenic switch. In particular, the callose-rich subintinal layer created immediately upon induction of embryogenesis was recently related to protection against stress. However, little is currently known about the functional significance of other compositional changes undergone by the walls of embryogenic microspores. We characterized these changes in *Brassica napus* at different stages during induction of embryogenic microspores and development of microspore-derived embryos (MDEs) by using a series of monoclonal antibodies specific for cell wall components, including arabinogalactan-proteins (AGPs), pectins, xyloglucan and xylan. We used JIM13, JIM8, JIM14 and JIM16 for AGPs, CCRC-M13, LM5, LM6, JIM7, JIM5 and LM7 for pectins, CCRC-M1 and LM15 for xyloglucan, and LM11 for xylan. By transmission electron microscopy and quantification of immunogold labeling on high-pressure frozen, freeze-substituted samples, we profiled the changes in cell wall ultrastructure and composition at the different stages of microspore embryogenesis. As a reference to compare with, we also studied *in vivo* microspores and maturing pollen grains. We showed that the cell wall of embryogenic microspores is a highly dynamic structure whose architecture, arrangement and composition changes dramatically as microspores undergo embryogenesis and then transform into MDEs. Upon induction, the composition of the preexisting microspore intine walls is remodeled, and unusual walls with a unique structure and composition are formed. Changes in AGP composition were related to developmental fate. In particular, AGPs containing the JIM13 epitope were massively excreted into the cell apoplast, and appeared associated to cell totipotency. According to the ultrastructure and the pectin and xyloglucan composition of these walls, we deduced that commitment to embryogenesis induces the formation of fragile, plastic and deformable cell walls, which allow for cell expansion and microspore growth. We also showed that these special walls are transient, since cell wall composition in microspore-derived embryos was completely different. Thus, once adopted the embryogenic developmental pathway and far from the effects of heat shock exposure, cell wall biosynthesis would approach the structure, composition and properties of conventional cell walls.

## Introduction

The plant kingdom is characterized by a high level of developmental plasticity and totipotency. Microspore embryogenesis is an androgenic form of totipotency in which haploid vacuolated microspores or young pollen grains are *in vitro* induced towards embryogenesis in the absence of fertilization, forming microspore-derived embryos (MDEs). Upon chromosome doubling, haploid embryos then transformed into doubled haploid (DH) and therefore fully homozygous plants in a single generation, placing this developmental switch in the center of numerous genetic and trait discovery breeding applications. Described more than 50 years ago, a deep mechanistic understanding of the changes undergone by induced microspores is still lacking.

In the model species *Brassica napus*, the androgenic switch is induced by a heat stress treatment of at least one day at 32°C (Lichter, 1982; Supena et al., 2008), after which some microspores/pollen change their developmental fate and form MDEs. Therefore, MDEs will simultaneously undergo considerable molecular and cellular modifications related to both heat stress exposure (the so-called heat stress response) and the onset of the embryogenic program. One of the most important modifications is a change in cytokinesis pattern and the formation of new cell walls. Induced microspores undergo symmetrical divisions similar to somatic cells (Zaki and Dickinson, 1991; Simmonds and Keller, 1999), instead of the asymmetrical pattern characteristic of the first pollen division. Cell walls are also different. After cytokinesis, the newly formed inner walls are irregular, incomplete, and present large deposits of cytoplasmic material secreted to the apoplast (Corral-Martínez et al., 2013; Parra-Vega et al., 2015a,b). Beneath their original intine, embryogenic microspores develop an additional layer, the subintinal layer, which is absent in non-embryogenic cultured microspores (Parra-Vega et al., 2015b; Rivas-Sendra et al., 2019). In addition to their structure, the composition of newly formed walls is also different from that of conventional cell walls. Primary cell walls are predominantly composed of cellulose, hemicellulose, pectin and structural glycoproteins (Cosgrove, 2005). In contrast, both subintinal layers and inner walls are callose-rich and cellulose-deficient during the first embryogenic stages (Parra-Vega et al., 2015b; Rivas-Sendra et al., 2019).

The deposition of other cell wall constituents has also been described as altered (Barany et al., 2010; El-Tantawy et al., 2013), but the extent and biological significance of these alterations are not yet clear. After cellulose, pectin is the principal carbohydrate in primary cell walls, being also present in secondary walls (Atmodjo et al., 2013). Pectin is involved in growth, morphogenesis, development, defense process, cell adhesion, wall porosity, and ion, enzyme and hormone binding (reviewed in Mohnen, 2008; Bou Daher and Braybrook, 2015). Among pectins, homogalacturonan (HG) is the predominant polymer, which is released to the cell wall in a highly methyl-esterified form (Willats et al., 2001). Then, HG is selectively modified, undergoing specific de-methyl-esterification by specific methyl esterases, depending on their final role (Micheli, 2001). Changes in methyl-esterification have also been described during *in vitro* morphogenic processes such as somatic (Chapman et al., 2000; Xu et al., 2011) and microspore embryogenesis (Barany et al., 2010; Solis et al., 2016), but we are still far from understanding their significance. Hemicellulose is another major component of primary walls. Hemicelluloses include xyloglucan and xylan, among others (Scheller and Ulvskov, 2010). Xyloglucan has a structural role, interacting with cellulose to form an extensive network, and playing a role in wall extensibility and cell expansion (Scheller and Ulvskov, 2010). In *arabidopsis*, its importance in cell wall formation was demonstrated by mutations in genes related to xyloglucan biosynthesis *in vivo* (Cavalier et al., 2008). During embryogenesis they have structural and also regulatory roles, participating in transduction of intercellular signals (Malinowski and Filipecki, 2002). Unlike xyloglucan, xylans are still poorly studied. They were initially associated with secondary cell walls, but recent studies revealed that they may also be present in primary walls (Faik, 2010; Mortimer et al., 2015).

In addition to polysaccharides, around 10% of the cell wall is composed of proteins. Among them, arabinogalactan proteins (AGPs) are a type of cell surface glycoproteins enriched in arabinose and galactose residues (Nguema-Ona et al., 2014). They appear to have a variety of roles beyond structural. Indeed, AGPs have been involved in a variety of biological processes such as cell growth, division and expansion, embryo pattern formation, modulation of cell wall mechanics or defense (Willats and Knox, 1996; Nothnagel, 1997; Cheung and Wu, 1999; Seifert and Roberts, 2007; Nguema-Ona et al., 2014). Specific roles have been proposed for AGPs in morphogenic processes such as zygotic (Pennell et al., 1991; Paire et al., 2003; Qin and Zhao, 2007), somatic (Pereira-Netto et al., 2007; Shu et al., 2014; Duchow et al., 2016) and microspore embryogenesis, where an effect in formation of the early embryogenic pattern (Tang et al., 2006) and in embryogenesis promotion has been documented in wheat, maize, *B. napus* and eggplant (Paire et al., 2003; Borderies et al., 2004; Letarte et al., 2006; Tang et al., 2006; Corral-Martínez and Seguí-Simarro, 2014). These facts led to the notion that perhaps, AGPs might be directly involved in embryogenesis induction (Seguí-Simarro et al., 2011). However, we are still far from understanding the precise role of AGPs in this process. Indeed, a deeper knowledge about the changes in these glycoproteins and the rest of cell wall components associated to microspore embryogenesis could help to enhance our understanding of this process, and of the role of cell wall in the embryogenic switch.

In a previous study, we conducted a detailed analysis of the changes in cell wall composition undergone by the different anther tissues at different stages during *B. napus in vivo* anther development (Corral-Martínez et al., 2016). Using the same battery of anti-AGPs, pectin, xyloglucan and xylan monoclonal antibodies, in this work we analyzed the changes in cell wall composition undergone *in vitro* cultured *B. napus* microspores/pollen as a consequence of their developmental switch towards embryogenesis. Thus, we studied embryogenic microspores and MDEs at different stages of their *in vitro* development. As references to compare with the different structures induced *in vitro*, we also studied *in vivo* microspores (still in the anther) at the suitable stage for embryogenesis induction, and *in vivo* maturing pollen grains. To study them, we produced and analyzed a collection of samples of *in vivo* and *in vitro* cultured cells processed for transmission electron microscopy (TEM) by high pressure freezing and freeze substitution (HPF-FS), acknowledged as the best procedure to maintain plant cell ultrastructure as close to the native state as possible during *in vivo* and *in vitro* processes (Gilkey and Staehelin, 1986; Corral-Martínez et al., 2013; Seguí-Simarro, 2015). In these samples we performed immunogold labeling with anti-AGPs, pectin, xyloglucan and xylan antibodies, and quantified the antibody signal to analyze differences in cell wall components. Our observations have relevant implications in the developmental fates of microspores undergoing them. This study adds to our previous studies focused on the ultrastructural changes occurring in the newly formed walls during the first stages of microspore embryogenesis (Parra-Vega et al., 2015b), and on the changes in calcium levels (Rivas-Sendra et al., 2017) and in callose and cellulose composition during induction of microspore embryogenesis (Rivas-Sendra et al., 2019). Together, these studies represent the most comprehensive and detailed work performed to date to investigate the changes in cell wall composition as a consequence of embryogenesis induction in *B. napus* microspores, and their implications in this developmental switch.

## Materials and Methods

### Plant Materials

*Brassica napus* L. donor plants of the highly embryogenic cv. Topas (DH4079) were grown in the greenhouses of the COMAV Institute (Universitat Politècnica de València, Valencia, Spain) under controlled temperature conditions and natural light during spring months.

### *B. napus* Microspore Culture

Flower buds containing mostly vacuolated microspores were selected as previously described (Custers, 2003). Briefly, buds were surface sterilized with 2% sodium hypochlorite for 10 min and washed three times in sterile distilled water. To release the microspores, buds were gently crushed in filter sterilized NLN-13 medium with the back of the plunger of a disposable 50 ml syringe. NLN-13 medium consists of NLN medium (Lichter, 1982) + 13% sucrose. The slurry was then filtered through 30 μm nylon cloths. The filtrate was transferred to 15 ml conical tubes and centrifuged at 800 rpm for 4 min at 800 rpm in a refrigerated Eppendorf Centrifuge 5804R with a 17.5 cm rotor radius. After discarding the supernatant, the pellet of microspores was resuspended in 10 ml of fresh NLN-13 medium. This procedure was repeated twice for a total of three centrifugations and resuspensions. Before the last centrifugation step, microspore concentration was calculated using a hemacytometer. The required volume of NLN-13 medium was added to adjust suspension to a concentration of 4 × 10^4^ microspores per ml. Adjusted microspore suspension was distributed in sterile culture dishes. Dishes were incubated in darkness for 24 h at 32°C to induce embryogenesis, and then continuously at 25°C for MDE progression up to the time of sample collection and processing.

### Processing of *B. napus* Materials for Transmission Electron Microscopy

For the *in vivo* materials, we processed anthers excised from 3.0 mm-long buds, containing microspores at the suitable stage for induction of embryogenesis (vacuolated microspores), and anthers excised from 3.7 mm-long buds, containing mid and mature pollen grains. They, together with *B. napus* microspore cultures, were processed as described in Seguí-Simarro (2015) and Corral-Martínez et al. (2016). In brief, anthers were excised, transferred to aluminum sample holders and cryoprotected with 150 mM sucrose. Cultured microspores and small MDEs were recovered from culture dishes by gently spinning culture media. Larger MDEs were manually picked up from cultures. These samples were transferred to aluminum sample holders and cryoprotected with their same glucose-rich culture medium. Then, both *in vivo* anthers and *in vitro* materials were high-pressure frozen in a Leica EM HPM-100 high-pressure freezer (Leica Microsystems, Vienna) and transferred to LN_2_. All samples were freeze substituted using a Leica AFS2 system (Leica Microsystems) with 2% OsO_4_ in anhydrous acetone at -80°C for 7 days, followed by slow warming to room temperature over a period of 2 days. After rinsing in several acetone washes, they were removed from the holders, incubated in propylene oxide for 30 min, rinsed again in acetone, and infiltrated with increasing concentrations of Epon resin (Ted Pella, Redding, CA) in acetone according to the following schedule: 4 h in 5% resin, 4 h in 10% resin, 12 h in 25% resin, and 24 h in 50, 75, and 100% resin, respectively. Polymerization was performed at 60° for 2 days. Six to eight resin blocks containing microspore cultures and MDEs at different developmental stages were obtained. Using a Leica UC6 ultramicrotome, thin sections (1 μm) were obtained for light microscopy observation, and ∼80 nm sections were obtained for electron microscopy. Sections were mounted on formvar-coated, 200 mesh copper grids, stained with uranyl acetate and lead citrate, and observed in a Jeol JEM 1010 electron microscope.

### Immunogold Labeling

For the detection of callose we used an anti-callose monoclonal antibody specifically recognizing linear (1→3)-β-oligosaccharide segments in (1→3)-β-glucans. For the detection of AGPs we used the following primary antibodies: JIM13, a rat IgM monoclonal antibody (mAb) recognizing the AGP2 epitope present in different plant exudates such as gum Arabic and gum ghatti (Knox et al., 1991); JIM8, a mAb (rat IgG2c) recognizing an AGP expressed during flower development in *B. napus* (Pennell et al., 1991); JIM14, a mAb (rat IgM) recognizing an AGP epitope different from that recognized by JIM13 (Knox et al., 1991); JIM16, a mAb (rat IgM) recognizing an AGP present in different plant exudates such as gum arabic, gum ghatti and gum karaya (Knox et al., 1991). For the detection of pectins we used the following antibodies: CCRC-M13, a mAb (mouse IgG1) specific for the pectic polymer rhamnogalacturonan-I (Pattathil et al., 2010); LM5, a mAb (rat IgG) crossreacting with (1-4)-β-D-galactosyl residues associated with rhamnogalacturonan-I (Jones et al., 1997); LM6, a mAb (rat IgG) crossreacting with (1-5)-α-L-arabinans (Willats et al., 1998); JIM7, a mAb (rat IgA) crossreacting with highly methyl-esterified epitopes of the homogalacturonan domain of pectic polysaccharides (Knox et al., 1990; Willats et al., 2000); JIM5, a mAb (rat IgG) crossreacting with low methyl-esterified epitopes of homogalacturonan (Knox et al., 1990; Willats et al., 2000); LM7, a mAb (rat IgM) crossreacting with partially methyl-esterified pectin homogalacturonan that results from non-blockwise de-esterification processes (Willats et al., 2001). For the detection of xyloglucan we used the following antibodies: CCRC-M1, a mAb (mouse IgG1) specific for galacto-fucosyalated xyloglucan (Pattathil et al., 2010); LM15, a mAb (rat IgG2c) crossreacting with the XXXG motif of xyloglucan (Marcus et al., 2008); For xylan detection we used LM11, a mAb (rat IgM) which recognizes arabinoxylan and low-substituted xylans (McCartney et al., 2005). For more information about the antibodies, see Table 1.

**Table 1:** Primary antibodies (ab) used for immunolocalization in this study.

Primary ab	Specificity	Provider	Dilution	Secondary ab
**Callose**		
Anti-callose	Callose	Biosupplies, Australia	1:5,000	Goat anti-mouse
**AGPs**		
JIM8	AGP	Carbosource, GA, United States	1:2	Goat anti-rat
JIM14	AGP	Carbosource, GA, United States	1:2	Goat anti-rat
JIM16	AGP	Carbosource, GA, United States	1:2	Goat anti-rat
JIM13	AGP	PlantProbes, Leeds, United Kingdom	1:5	Goat anti-rat
**Pectins**		
CCRC-M13	Rhamnogalacturonan-I	Carbosource, GA, United States	1:2	Goat anti-mouse
LM5	(1-4)-β-D-galactan	PlantProbes, Leeds, United Kingdom	undiluted	Goat anti- rat
LM6	(1-5)-α-L-arabinans	PlantProbes, Leeds, United Kingdom	undiluted	Goat anti- rat
JIM7	Highly methyl-esterified homogalacturonans	PlantProbes, Leeds, United Kingdom	1:2	Goat anti- rat
JIM5	Low methyl-esterified homogalacturonans	PlantProbes, Leeds, United Kingdom	1:2	Goat anti- rat
LM7	Partially methyl-esterified homogalacturonans	PlantProbes, Leeds, United Kingdom	undiluted	Goat anti- rat
**Hemicelluloses**		
CCRC-M1	Fucosylated xyloglucan	Carbosource, GA, United States	1:2	Goat anti-mouse
LM15	XXXG Xyloglucan	PlantProbes, Leeds, United Kingdom	undiluted	Goat anti- rat
LM11	Arabinoxylan and low-substituted xylan	PlantProbes, Leeds, United Kingdom	undiluted	Goat anti- rat


Immunogold labeling was performed in HPF-fixed, OsO_4_-treated, epoxy-embedded samples. This processing is not compatible with immunolocalization of protein epitopes, because OsO_4_ is likely to alter protein epitopes. However, we demonstrated that this procedure can be successfully used to specifically detect carbohydrate epitopes (Parra-Vega et al., 2015b; Corral-Martínez et al., 2016), not so sensitive to OsO_4_. Thus, by using this approach, we were able to combine specific immunolabeling with excellent ultrastructural preservation. For immunolocalization of cell wall components, we deposited Epon sections (80–100 nm) onto Formvar and carbon-coated, 200-mesh nickel grids (Electron Microscopy Sciences). Sections were hydrated with PBS during 1 min. Non-specific binding was avoided by applying PBS + 0.2% BSA + 3% skimmed milk for 30 min. Afterwards sections were incubated with the corresponding primary antibody diluted according to Table 1 in PBS + 0.2% BSA (1% BSA for the anti-callose antibody) for 1 h at 25°C. Next, sections were washed with PBS + 0.2% BSA six times for 5 min each, and incubated with the corresponding secondary antibodies (see Table 1) conjugated with 10 nm colloidal gold (all from BBI Solutions, United Kingdom) diluted 1:25 in PBS + 0.2% BSA (1% BSA for the anti-callose antibody) for 45 min at 25°C. After five 5-min washes with PBS, sections were post-fixed with 2% glutaraldehyde in PBS for 10 min, washed with PBS and distilled water, and stained with uranyl acetate in 70% methanol (6 min) and lead citrate (30 s). Controls were performed by omitting primary antibodies to check non-specific binding of the secondary antibody.

### Quantification of Immunogold Labeling

From each developmental stage, three different blocks were randomly selected and micrographs were taken systematically always at the same magnification. Sampling was carried out over a number of micrographs taken randomly from all the cells of interest on each grid. The minimum number of micrographs was determined using the progressive mean test (Williams, 1977), with a minimum confidence limit of α = 0.05. In general, the micrograph number revolved around 15-20 per antibody and developmental stage studied. Labeling density was defined as the number of particles per area unit (μm^2^). This number of particles was determined by hand counting particles over the compartments under study. These compartments, determined *a priori*, were (1) the cytoplasmic Golgi stacks and vesicles, (2) the cell wall, differentiating between middle lamella and the rest of the cell wall when differences between both regions were detected, (3) the intine for microspores and pollen, (4) the inner walls in embryogenic microspores and MDEs; and (5) the epidermal walls in MDES. As an estimation of background noise, we determined the particle labeling density over regions where the presence of the studied carbohydrates is not expected. These regions included the cytoplasm (excluding the Golgi stacks and Golgi-derived vesicles), cytoplasmic organelles and nucleus. Background noise never exceeded 5%, compared to the quantified signal. The area in μm^2^ was measured using a square lattice composed of 11 × 16 squares of 15 × 15 mm each.

Initially, we considered the following stages/cell types for our study: *in vivo* microspores, *in vivo* pollen, first dividing embryogenic microspores, 2-4 celled embryogenic structures, multicellular structures, defined as structures with 6–10 cells, pollen-like structures, globular MDEs differentiating between the embryo proper and the suspensor domains, heart-shaped, torpedo and cotyledonary MDEs. Specific differences among these stages were found for JIM13 immunogold labeling. However, the rest of antibodies showed comparable results for embryogenic microspores from the 2 to the 10 cell stages, and also for the embryo proper and suspensor domains of the globular MDE. In these cases, we grouped together the comparable stages for simplicity. For each stage and tissue, labeling density (number of gold particles/μm^2^) was expressed as the mean labeling density of all micrographs ± SD. Comparisons between mean labeling densities among stages and among tissue and subcellular regions were performed by one-way ANOVA tests using StatGraphics software. Means were separated using a Tukey’s test with *p* ≤ 0.05. Labeling densities and statistical comparisons were shown as independent histograms for each antibody and tissue. In order to facilitate comparisons between tissues, all the histograms were built using the same Y-axis scale, although in some cases, this led to the occurrence of empty or almost empty charts.

## Results

**FIGURE 1:**
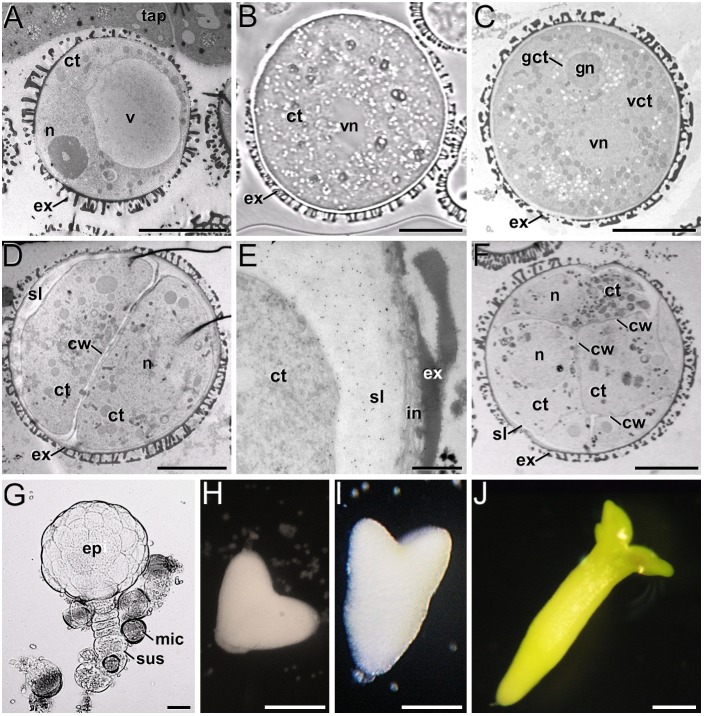
*B. napus in vivo* microspores and pollen, and development of microspore cultures. **(A)** *In vivo* vacuolated microspore, still within the anther locule and surrounded by tapetum (tap). **(B)** *In vitro* pollen-like structure. The only nucleus visible in this section is the vegetative nucleus (vn). **(C)** *In vivo* pollen grain. Note the generative nucleus (gn) within the cytoplasm of the generative cell (gct), and the vegetative nucleus (vn), all embedded within the cytoplasm of the vegetative cell (vct). **(D)** 2-celled embryogenic microspore, with a newly formed inner cell wall (cw) and a thick subintinal layer (sl). **(E)** Anti-callose immunogold labeling in a 2-celled embryogenic microspore, revealing the abundance of callose in the subintinal layer. **(F)** Multicellular structure of around 8-10 cells. **(G)** Suspensor-bearing globular MDE surrounded by dead or arrested microspores (mic), and showing the embryo proper (ep) and suspensor (sus) domains. **(H)** Heart-shaped MDE. **(I)** Torpedo MDE. **(J)** Cotyledonary MDE. ct: cytoplasm; ex: exine; n: nucleus; v: vacuole. Bars: **(A–D)**: 10 μm; **(E)**: 500 nm; **(F)**: 10 μm; **(G)**: 20 μm; **(H–J)**: 200 μm.

In the *B. napus* DH4079 microspore culture system, fresh (*in vivo*) vacuolated microspores (Figure 1A) are isolated from anthers and *in vitro* inoculated. After the heat shock-based induction treatment, many of them did not survive and died. Other microspores, not sensitive to the inductive treatment, followed a gametophytic-like program, becoming pollen-like structures (Figure 1B), structurally very similar to *in vivo* mature pollen grains (Figure 1C). Microspores sensitive to the inductive treatment were effectively reprogrammed towards embryogenesis, reabsorbing the large vacuole and dividing to form a thick inner wall (Figure 1D). In addition, embryogenic microspores developed a thick, continuous subintinal layer that separated the protoplast from the original microspore walls. Anti-callose immunogold labeling revealed that the subintinal layer was rich in callose (Figure 1E). Two-to-four-celled embryogenic structures could be observed 3–4 days after inoculation (Figure 1D), and multicellular structures (around 8–10 cells) were found in 6–7-day old cultures (Figure 1F). From this stage on, the different growth rates of each individual progressively increased the heterogeneity of cultures, but in general, globular (Figure 1G) and heart-shaped MDEs (Figure 1H) could be found after 7–12 days of culture, and torpedo (Figure 1I) and cotyledonary MDEs (Figure 1J) were observed only after 2–3 weeks of culture. Each of these stages and developmental fates showed defined patterns of AGP, pectin, xyloglucan and xylan epitope distribution, as described next.

### Levels and Distribution of AGP Epitopes

**FIGURE 2:**
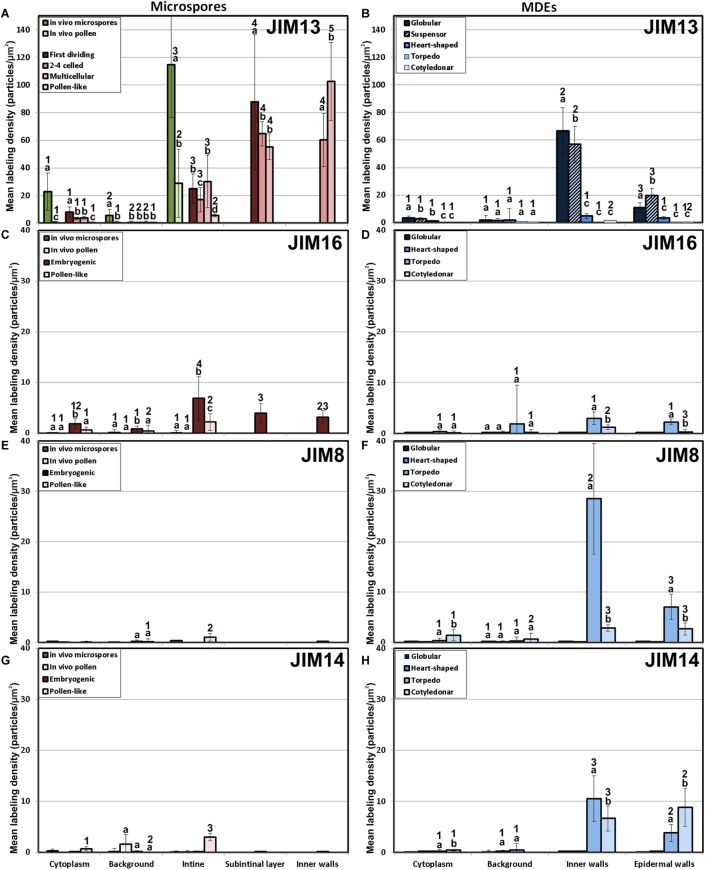
Quantification of AGP immunogold labeling with JIM13 **(A,B)**, JIM16 **(C,D)**, JIM18 **(E,F)** and JIM14 **(G,H)** in *in vivo* microspores and pollen grains, and during microspore embryogenesis and MDE development. Quantification is expressed as mean labeling density in the Y-axis. For each subcellular region, different letters indicate significant differences in mean labeling density among developmental stages. For each developmental stage, different numbers indicate significant differences among subcellular regions.

**FIGURE 3:**
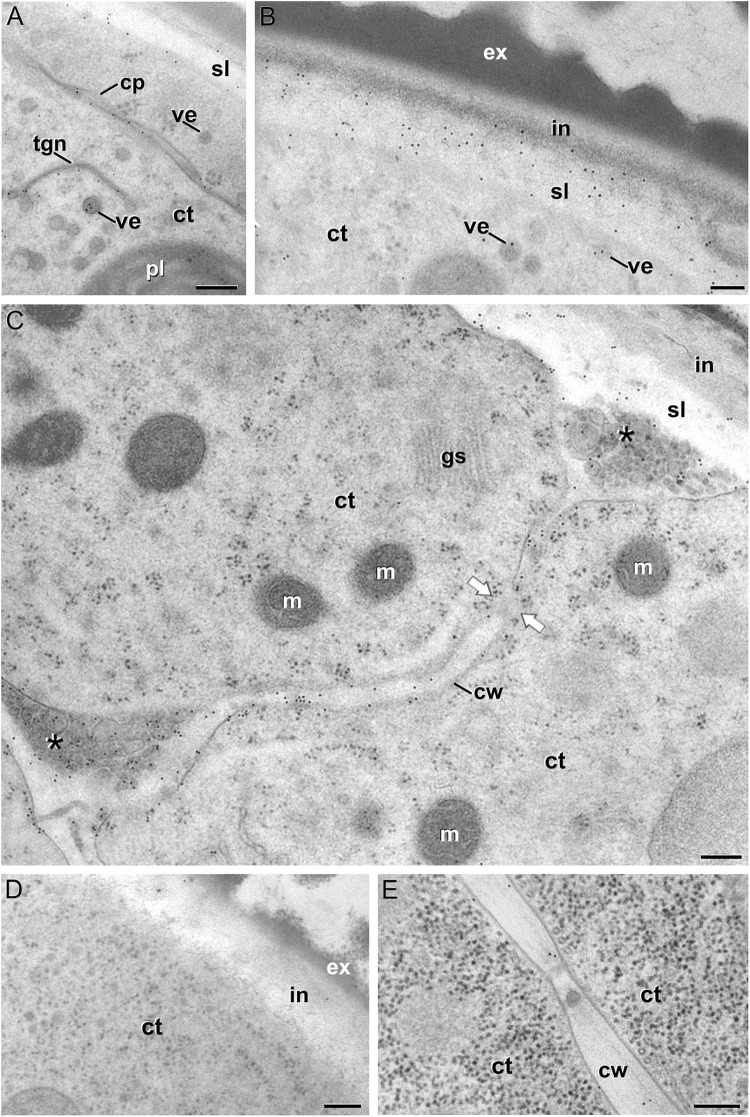
JIM13 immunogold labeling. **(A–C)**: Images of dividing embryogenic microspores, showing details of a maturing cell plate (cp) in **(A)**, the intine (in) and subintinal layer (sl) in **(B)**, and a fragmented cell wall (cw) with gaps (white arrows) and deposits of cytoplasmic material (asterisks) secreted to the apoplast in **(C)**. **(D)** Pollen-like structure. **(E)** Torpedo MDE. ct: cytoplasm; ex: exine; gs: Golgi stack; m: mitochondrion; pl: plastid; tgn: trans-Golgi network; ve: vesicle. Bars: 100 nm.

We previously demonstrated that the subintinal layer and the first cell walls of embryogenic microspores are compositionally different from the intine of pollen-like structures and from conventional cell walls of mature MDEs in terms of callose and cellulose content (Parra-Vega et al., 2015b; Rivas-Sendra et al., 2019). Based on this, we herein investigated whether these differences are extensive to other cell wall components. First, we performed immunogold labeling with anti-AGP antibodies specific for different AGP epitopes (see Table 1) and quantified the labeling. Among them, the most abundant by far was the epitope crossreacting with JIM13. Immunogold labeling with JIM13 antibodies was found in nearly all the stages and subcellular regions studied, both *in vivo* and *in vitro* (Figure 2). However, the highest labeling densities were by far those of the intine of *in vivo* microspores and the subintinal layer and inner walls of embryogenic microspores. Labeling was also present in the intine of *in vivo* pollen grains and *in vitro* embryogenic microspores, but at levels considerably lower than in the subintinal layer. In the cytoplasm, JIM13 antibodies labeled the Golgi stacks, trans-Golgi network (TGN), the vesicles produced by fragmentation of the TGN and, in embryogenic microspores, the growing cell plate (Figure 3A), clearly tracing the route followed by AGPs from their synthesis in the Golgi cisternae to their deposition into the cell plate. As embryogenic microspores underwent more rounds of division, the labeling density in cytoplasmic vesicles and Golgi stacks decreased progressively, paralleling that of the subintinal layer (Figure 2A, 3B). The newly made, inner cell walls showed abundant and increasing JIM13 labeling (Figure 3C), reaching a maximum in multicellular microspores (Figure 2A). Gold particles intensely decorated the deposits of cytoplasmic materials and vesicular elements (asterisks in Figure 3C) secreted to the apoplast as described by Corral-Martínez et al. (2013). This indicated that the secreted cytoplasmic material contained AGPs at the time of its delivery and, therefore, secretion of these compounds is likely to occur simultaneously. In contrast to the abundant anti-AGP labeling found in the intine of *in vivo* microspores and the subintinal layer and cell walls of embryogenic microspores, the thickened intine of pollen-like structures (Figure 3D) showed very scarce labeling (Figure 2A). During MDE development, we observed a decrease (with respect to multicellular microspores) of the JIM13 epitopes detected in inner cell walls of both the suspensor and the embryo proper domains (Figure 2B). However, the most dramatic decrease was measured after the globular-to heart-shaped MDE transition, the signal of heart-shaped, torpedo (Figure 3E) and cotyledonar MDEs being very scarce or undetectable. In the MDE epidermal layer, the levels of JIM13 epitopes were always lower than in inner walls.

As to JIM16, JIM8 and JIM14 epitopes, they were found at levels dramatically lower than JIM13 epitopes in all the stages and subcellular compartments, being undetectable (signal not significantly different from background) in many cases, including the two *in vivo* stages studied (Figure 2). Since no differences were found during the first divisions of the embryogenic microspores, for the rest of immunolocalization data we opted for a simplification of charts, presenting only the results of the *in vivo* stages, embryogenic microspores and pollen-like grains. JIM16 epitopes were clearly predominant in embryogenic microspores, and principally in the intine, followed by the subintinal layer and inner walls. However, their levels were low, never exceeding 10 particles/μm^2^ (Figure 2C). During embryogenesis, their levels were never higher than background signal (Figure 2D). JIM8 epitopes were nearly undetectable in all stages and regions except for the inner cell walls and epidermis of torpedo and cotyledonar embryos, where particle density reached relatively high levels (Figure 2E,F). Although JIM14 epitopes presented detectable levels in the intine of *in vivo* pollen and *in vitro* pollen-like grains (Figure 2G), the general pattern was similar to that obtained with JIM8, with higher particle densities only in the inner cell walls and epidermis of torpedo and cotyledonar embryos (Figure 2H).

Together, these results demonstrated that the AGP composition of the cell walls produced during microspore embryogenesis is unique, remarkably different from *in vivo* microspore/pollen development, and highly dynamic, changing dramatically as the microspore becomes embryogenic and then develops as an MDE. However, among the AGP epitopes studied, the most abundant by far were those recognized by JIM13, which accumulated principally in walls of *in vivo* microspores and *in vitro* embryogenic microspores and MDEs during the first stages of their development.

### Levels and Distribution of Pectin Epitopes

**FIGURE 4:**
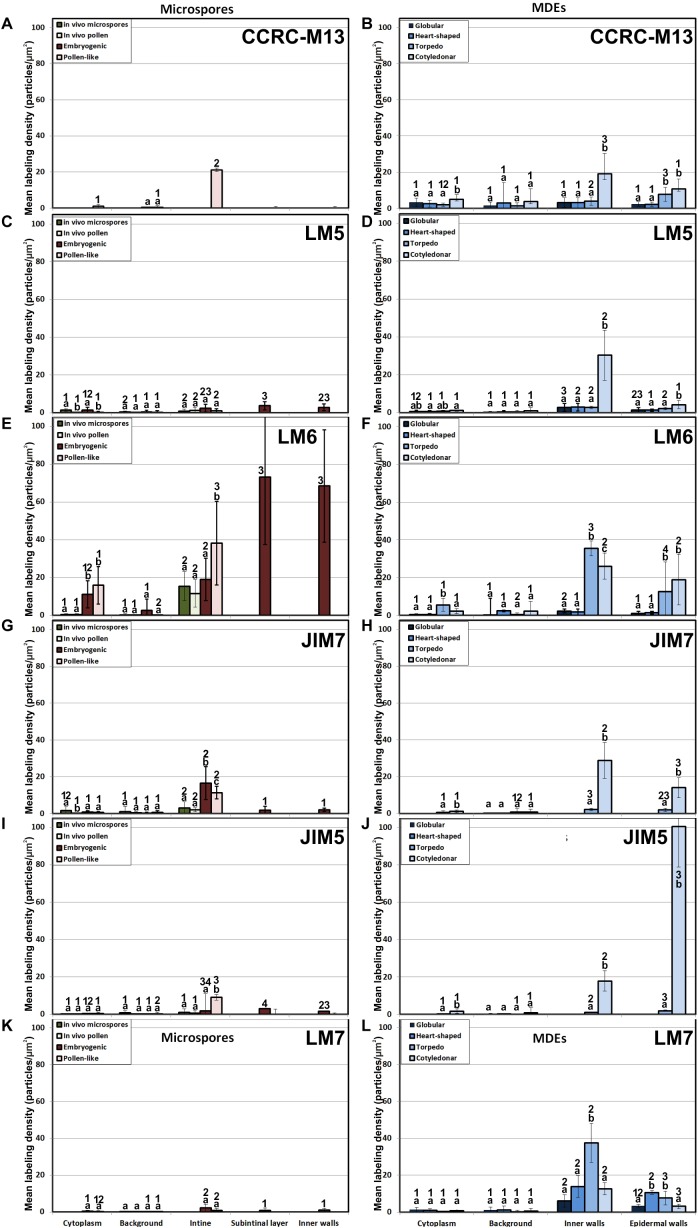
Quantification of pectin immunogold labeling with CCRC-M13 **(A,B)**, LM5**(C,D)**, LM6 **(E,F)**, JIM7 **(G,H)**, JIM5 **(I,J)** and LM7 **(K,L)** in *in vivo* microspores and pollen grains, and during microspore embryogenesis and MDE development. Quantification is expressed as mean labeling density in the Y-axis. For each subcellular region, different letters indicate significant differences in mean labeling density among developmental stages. For each developmental stage, different numbers indicate significant differences among subcellular regions.

The epitopes recognized by CCRC-M13 (rhamnogalacturonan-I) were almost undetectable in any compartment of *in vivo* microspores and pollen, and of embryogenic microspores, but clearly found in the intine of *in vitro* pollen-like grains (Figure 4A). During MDE development, these epitopes were detected at levels progressively higher in inner and epidermal walls, with maximal accumulation in cotyledonary MDEs (Figure 4B).

**FIGURE 5:**
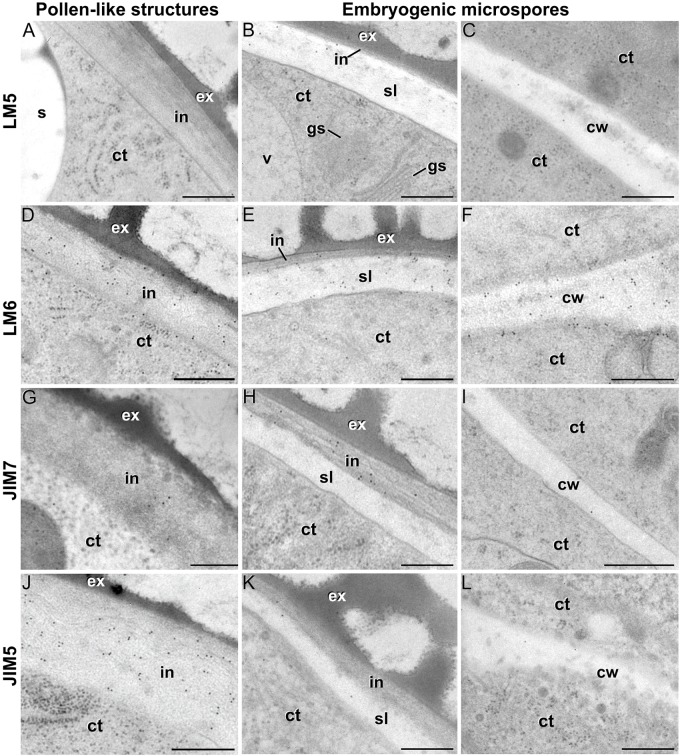
Pectin immunogold labeling with LM5 **(A–C)**, LM6 **(D–F)**, JIM7 **(G–I)** and LM5 antibodies **(J–L)** in the cytoplasm and coat of pollen-like structures (left column) and embryogenic microspores (central column), and in inner cell walls of embryogenic microspores (right column). ct: cytoplasm; cw: cell wall; ex: exine; gs: Golgi stack; in: intine; s: starch granule; sl; subintinal layer; v: vacuole. Bars: 100 nm.

**FIGURE 6:**
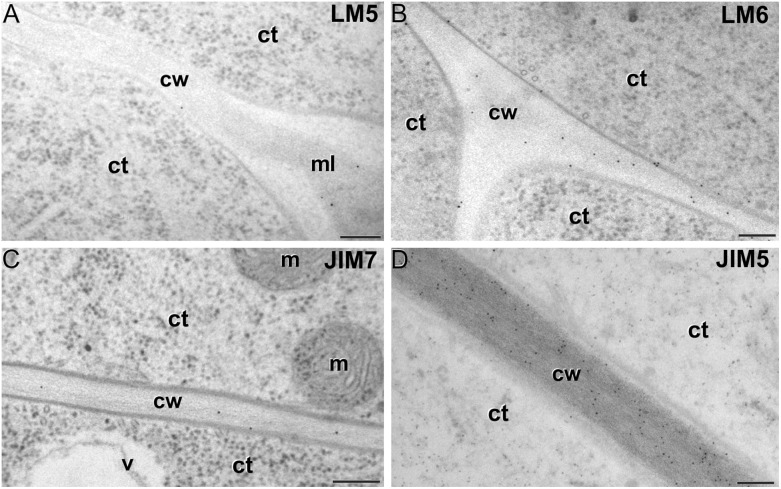
Pectin immunogold labeling with LM5 **(A)**, LM6 **(B)**, JIM7 **(C)** and LM5 antibodies **(D)** in cotyledonar MDEs. ct: cytoplasm; cw: cell wall; m: mitochondrion; v: vacuole. Bars: 100 nm.

LM5 epitopes ((1-4)-β-D-galactan residues from rhamnogalacturonan-I) were in general very scarcely found (Figure 4C). Very low labeling densities, but significantly higher than background, were measured in cell walls of pollen-like structures (Figure 5A) and embryogenic microspores, at both the subintinal layer (Figure 5B) and the newly formed first cell walls (Figure 5C). In inner walls, LM5 epitopes were found dispersed in an electron translucent and disorganized matrix. During MDE development (Figure 4D), the levels of these epitopes increased only in cotyledonar MDEs, in the inner walls and, to a much lower extent, in the epidermal walls. In these two cases, gold particles were found principally in the middle lamella formed between two adjacent cells (Figure 6A), as expected for the localization of unesterified pectins (Cosgrove, 1997).

LM6 epitopes ((1–5)-α-L-arabinans) were abundantly found in the thickened intine of pollen-like structures (Figure 5D) and, to a lower extent, in the thin intine of embryogenic microspores (Figure 5E), but not of *in vivo* micropores and pollen grains (data not shown). Indeed, the labeling density of the intine of pollen-like structures was nearly twice that of embryogenic microspores (Figure 4E). However, the highest densities were found in the subintinal layer (Figure 5E) and inner walls (Figure 5F) of embryogenic microspores. Young MDEs presented very low levels at all regions, but torpedo and cotyledonar MDEs showed increased LM6 signal in the inner and epidermal walls (Figure 4F). In inner cell walls, labeling was found principally in the middle lamella (Figure 6B). According to manufacturer specifications, LM6 has no cross-reactivity with gum Arabic, but it may recognize AGPs in some species. However, the labeling pattern described herein for LM6 is strikingly different from those of the anti-AGP antibodies shown in Figure 2, which makes it reasonable to assume that at least in *B. napus*, the LM6 mAb is not recognizing any of the AGPs crossreacting with the anti-AGP JIM antibodies, and likely, any other AGP.

As to JIM7 epitopes (highly methyl-esterified pectins; Figure 4G,H), they were found to accumulate at levels higher than background only in the intine, more in *in vitro* than in *in vivo* structures, and with levels in pollen-like structures (Figure 5G) lower than in embryogenic microspores (Figure 5H). In the latter, the subintinal layer (Figure 5H) and inner cell walls (Figure 5I) showed almost no labeling. Signal was found at moderate levels in walls of cotyledonar MDEs (Figure 6C). JIM5 epitopes (low methyl-esterified pectins; Figure 4I,J) presented a remarkably similar pattern, but no significant signal was measured in *in vivo* structures, and signal in the intine of pollen-like structures (Figure 5J) was much more abundant than in embryogenic microspores (Figure 5K). Almost no signal was found in the inner cell walls of embryogenic microspores (Figure 5L). The highest levels were observed in inner (Figure 6D) and epidermal walls of cotyledonar MDEs.

LM7 epitopes (partially methyl-esterified pectins) presented very low levels in *in vivo* structures and during the first stages of *in vitro* embryogenesis, with significant accumulation only in the intine of both embryogenic microspores and pollen-like structures (Figure 4K). However, MDE development was characterized by a progressive accumulation of these epitopes in inner and epidermal walls, reaching a peak at the torpedo stage and then decreasing at the cotyledonar stage (Figure 4L).

Based on these results we concluded that, in terms of pectin composition, the intine of *in vivo* and *in vitro* (embryogenic) microspores was similar, but it differed between *in vivo* and *in vitro* pollen grains. We also deduced that the pectin levels of the intine differed in pollen-like and embryogenic microspores. In addition, the first cell walls formed in embryogenic microspores were dramatically different from MDE walls. In embryogenic microspores, the most abundantly detected pectins were those containing arabinan. During MDE development, pectin deposition seemed to be highly dynamic, each of the pectin epitopes studied having a specific temporal and spatial pattern of deposition.

### Levels and Distribution of Xyloglucan and Xylan Epitopes

**FIGURE 7:**
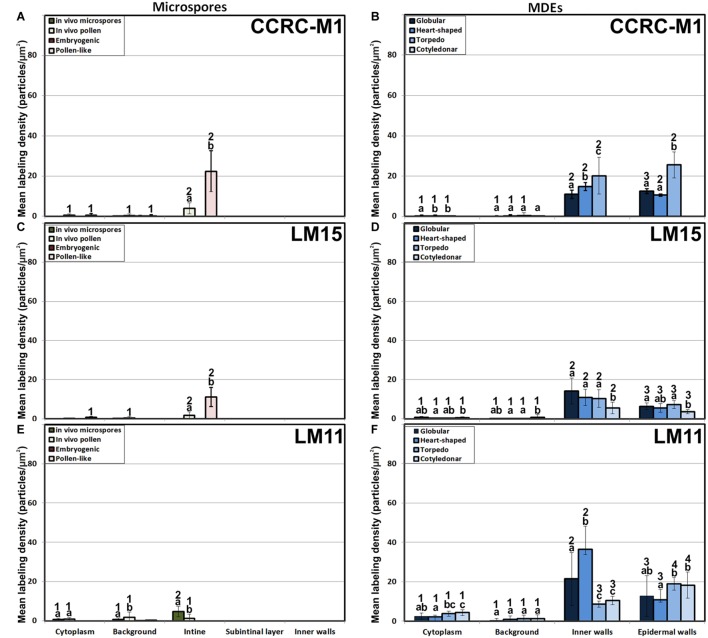
Quantification of xyloglucan and xylan immunogold labeling with CCRC-M1**(A,B)**, LM15 **(C,D)** and LM11 **(E,F)** in *in vivo* microspores and pollen grains, and during microspore embryogenesis and MDE development. Quantification is expressed as mean labeling density in the Y-axis. For each subcellular region, different letters indicate significant differences in mean labeling density among developmental stages. For each developmental stage, different numbers indicate significant differences among subcellular regions.

**FIGURE 8:**
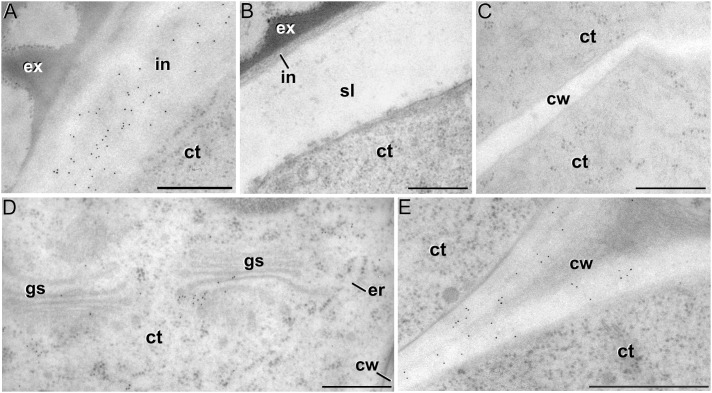
Xyloglucan immunogold labeling with the mAb CCRC-M1. **(A)** Detail of the intine (in) in a pollen-like structure. **(B)** Detail of the intine and subintinal layer (sl) of an embryogenic microspore. Note that the intine wall is strongly labeled in pollen-like structures **(A)**, but not in embryogenic microspores **(B)**. **(C)** Inner cell wall (cw) of an embryogenic microspore. **(D,E)** Torpedo MDEs. Details of the cytoplasm (ct) are shown in **(D)**, where cytoplasmic gold particles strongly decorate the Golgi stacks (gs) and associated vesicles. The cell wall is shown in **(E)**. ex: exine; er: endoplasmic reticulum. Bars: 500 nm.

The antibody CCRC-M1 (Figure 7A) detected significant amounts of fucosylated xyloglucan epitopes only in the intine of *in vivo* pollen grains and *in vitro* pollen-like structures (Figure 8A). No epitopes were detected in *in vivo* microspores nor in early embryogenic structures, neither in the subintinal layer (Figure 8B) nor in inner cell walls (Figure 8C). The anti-xyloglucan antibody LM15 (Figure 7C) exhibited a remarkably similar pattern, with only small quantitative differences. However, the labeling patterns of anti-xyloglucan antibodies differed notably during MDE development. Although in all cases labeling was observed in Golgi stacks (Figure 8D), as well as in inner (Figure 8E) and epidermal MDE walls, the temporal pattern was different. CCRC-M1 epitopes were detected at all stages but cotyledonar, with levels progressively higher as the MDE proceeded in development, reaching the highest in torpedo MDEs (Figure 7B). In contrast, LM15 epitopes showed an opposite temporal pattern, with the highest levels at the globular MDE stage, and then progressively decreasing as the MDE matures (Figure 7D). As for the anti-xylan LM11 antibody, it only detected significant signal of arabinoxylan and low-substituted xylan epitopes in the intine of *in vivo* microspores, but not in any *in vitro* cultured cell during the first stages (Figure 7E). Once embryogenic microspores were transformed into MDEs, xylan epitopes were found at all MDE stages, although they were mostly expressed in heart-shaped MDEs (Figure 7F).

In summary, during the first stages of microspore culture, xyloglucan but not xylan specifically accumulated in the intine of *in vivo* and *in vitro* pollen grains, while they were absent from all the walls of *in vivo* and embryogenic microspores, which revealed a notable difference in the intine of these two types of structures. Xyloglucan seemed specific for pollen or pollen-like development, whereas xylan seemed specific for *in vivo* microspores. During MDE development, both xyloglucan and xylan were deposited in the inner and epidermal walls. However, each xyloglucan and xylan epitope showed a specific temporal and spatial pattern of deposition.

## Discussion

In this study, we showed that the change of developmental fate towards microspore embryogenesis determines (1) the formation of new walls with a unique structure and composition, and (2) remodeling of the existing microspore intine. One of the first signs of embryogenesis induction is the *de novo* assembly of a callose-rich and cellulose-deficient subintinal layer and inner wall separating both daughter cells (Parra-Vega et al., 2015b). We herein extended the characterization to their pectin, xyloglucan and xylan components, showing that these *de novo*-formed walls have a special composition, different from the intine previously formed *in vivo*. We also demonstrated that induction of embryogenesis affects intine composition. A comparison between *in vivo* microspores and *in vitro*, just induced embryogenic microspores reveals that they are ultrastructurally similar and have comparable levels of xyloglucan, xylan (very low/absent), and pectin epitopes, with the exception of JIM7 epitopes (highly methyl-esterified HG). However, both intines are remarkably different in terms of AGP epitopes. Thus, it seems that the change in developmental fate implies the formation of new walls and the delivery of particular AGPs to both the existing intine and the newly formed inner walls and subintinal layer. This is in line with other observations of different AGP profiles in somatic cells of the same species but with different *in vivo* and *in vitro* developmental fates (Immerzeel et al., 2004; Wisniewska and Majewska-Sawka, 2008), and will have a reflection in the properties of these cell walls, as discussed in the next sections.

### The *in vitro* Environment Accounts for Changes in Pectin and Xyloglucan Composition of Differentiated Cells

The intine is supposed to have a pectocellulosic nature (Sitte, 1953). Thus, one could expect similar intine walls in cells similarly committed to gametogenesis. Ultrastructurally, we did not find differences between *in vivo* maturing pollen grains and *in vitro* pollen-like grains (compare Figure 1B,C). However, remarkable quantitative differences in composition for LM6, JIM7 and JIM5 pectin epitopes were found (Figure 4), As to xyloglucan, LM15 (specific for the xyloglucan backbone) and CCRC-M1 (detecting fucosylated xyloglucan), showed preferential accumulation in the intine of pollen-like structures, whereas *in vivo* pollen grains showed very low levels (Figure 7). Only LM5 (1-4 β-D-galactan) epitopes showed comparable (low) levels. These differentiated structures have the same origin (microspores) and similar developmental fates. Their only difference is their environment. Whereas pollen grains develop *in anthero*, pollen-like grains are exposed to *in vitro* culture conditions, which alter the normal cell wall biosynthesis of these cells. Consequently, *in vitro* culture *per se* should be taken into account as a factor to explain cell wall changes.

### AGPs With JIM13 Epitopes Are Associated With Cell Totipotency

Among the AGP epitopes studied, JIM13 epitopes were by far the most abundant. According to manufacturer specifications, JIM13 may in some cases recognize some rhamnogalacturonan I epitopes. However, the labeling pattern described herein for JIM13 (Figure 2A,B) is strikingly different from that of CCRC-M13, specific for rhamnogalacturonan-I (Figure 4A,B). Thus, it seems reasonable to consider that at least for our study in *B. napus*, JIM13 mAbs are not recognizing rhamnogalacturonan I epitopes. JIM13 antibodies accumulated in the intine, the subintinal layer and in inner cell walls. During *in vivo* anther development, high levels of JIM13 labeling were found in microspores. It was previously proposed that the tapetum, together with other anther layers, would account for the high levels of JIM13 epitopes found in microspores during anther development (Corral-Martínez et al., 2016). *In vitro*, JIM13 antibodies strongly decorated the massive extracytoplasmic deposits previously identified as partially digested cytoplasmic material transported to the apoplast as a consequence of embryogenesis induction (Corral-Martínez et al., 2013). This indicates that, in parallel to conventional transport pathways via vesicles, AGPs with JIM13 epitopes would be massively excreted by this mechanism, accounting for the high levels of this epitope detected in embryogenic microspores and young MDEs. In contrast, JIM13 epitopes were very scarcely detected in Golgi stacks, vesicles and intine of pollen grains (where this epitope is restricted to the still totipotent generative cell wall; Corral-Martínez et al., 2016), pollen-like grains and older MDE stages, where no extracytoplasmic deposits have been described. Considering that the highest levels of JIM13 epitopes are specifically found in totipotent cells such as *in vivo* microspores, generative cells and *in vitro* just induced embryogenic microspores and early globular embryos (Figure 2), but not in differentiated cells of *in vivo* pollen grains, *in vitro* pollen-like grains or maturing MDEs, it seems clear that the presence of AGPs containing JIM13 epitopes is tightly associated to totipotency and proliferation. This is consistent with a large number of studies on different species that attribute AGPs in general, and JIM13 in particular, a role in the promotion of zygotic (Pennell et al., 1991; Paire et al., 2003; Qin and Zhao, 2007), somatic (Pereira-Netto et al., 2007) and microspore embryogenesis (Borderies et al., 2004; Tang et al., 2006), and relate the exogenous application of AGPs to increased embryogenic competence of somatic cells (Feher et al., 2003) and microspores (Paire et al., 2003; Letarte et al., 2006; Corral-Martínez and Seguí-Simarro, 2014; Makowska et al., 2017).

As to the role of AGPs in microspore embryogenesis, it has been thought for long that they could act as signaling/regulatory molecules (Pereira et al., 2015) but, surprisingly, no hints about their putative signaling mechanism have been reported so far. However, recent studies point to a radically different role as calcium capacitors. It is known that periplasmic AGPs act as a capacitor that serves as a source of cytosolic Ca^2+^, thus regulating the Ca^2+^ discharges needed for many plant growth and developmental processes (Lamport and Varnai, 2013). During *in vivo* development, there is a clear increase in cytosolic Ca^2+^ contents in vacuolated microspores and young pollen grains, which are the stages suitable for embryogenesis induction (Rivas-Sendra et al., 2017), and the stages where we found the highest levels of JIM13 epitopes. After *in vitro* induction, Ca^2+^ levels increased even more only in embryogenic microspores (Rivas-Sendra et al., 2017, 2019), the *in vitro* stage with highest levels of JIM13 epitopes. Ca^2+^ was found to be responsible for the embryogenic competence of microspores that are able to form a callosic, osmoprotective subintinal layer (Rivas-Sendra et al., 2019). As seen, there is a clear relationship between totipotency, embryogenic competence, Ca^2+^ increases and high levels of JIM13 epitopes in the microspore walls. Since the main elements of the Lamport and Varnai (2013) model are present in embryogenic microspores, it seems reasonable to envisage that the high levels of JIM13-crossreacting AGPs of embryogenic microspores could act, according to such model, as Ca^2+^ capacitors to provide the high Ca^2+^ levels needed to induce the embryogenic response.

### The Pectin and Xyloglucan Profiles of Embryogenic Microspores Produce Weak and Plastic Walls, Reflecting the Commitment to Microspore Embryogenesis

It is widely accepted that the chemical composition of plant cell walls is tightly related to their structure, adherence, and mechanical and rheological properties, which in turn are a reflection of cell function and fate (Willats et al., 2001; Harholt et al., 2010). In this context, pectin side chains act as plasticizers in cell walls that undergo large physical remodeling. Thus, walls of differentiating/elongating cells have abundant galactan-rich pectins (Willats et al., 1999; McCartney et al., 2003; Majewska-Sawka et al., 2004; Harholt et al., 2010) that provide them increased wall firmness (McCartney et al., 2000). In turn, walls of proliferating cells, both *in vivo* and *in vitro*, typically have very low levels of galactan residues and are rich in arabinan residues (Kikuchi et al., 1995; Willats et al., 1999; Majewska-Sawka and Münster, 2003), which confers them the plasticity needed for cell division and growth. In the intine of totipotent vacuolated microspores, the *in vivo* levels of galactan residues were low, whereas those of arabinans were more than 10x higher (Figure 4). Upon induction to embryogenesis, the intine of proliferating embryogenic microspores presented comparable levels, but newly formed subintinal layers and inner walls presented dramatically higher levels of arabinans. According to Harholt et al. (2010), arabinans keep adjacent HG domains separated, preventing their crosslinking for increased wall rigidity. Therefore, the arabinan-rich, newly formed walls of embryogenic microspores will be plastic and flexible. Xyloglucan is known to bind cellulose microfibrils, whereas fucose residues are important for the establishment of xyloglucan-cellulose interactions that provide tensile strength (Peña et al., 2004). Interestingly, embryogenic microspore walls are devoid of fucosylated xyloglucan (Figure 7) and cellulose (Parra-Vega et al., 2015b; Rivas-Sendra et al., 2019) which makes such interactions impossible. Therefore, this profile will also account for weak, breakable walls.

Aside of plasticity and firmess, the pectin profile and level of methylation have also been demonstrated as key in cell adhesion (Willats et al., 2001). Pectin enriched in LM7 and JIM5 epitopes are key to maintain cell wall adhesion. In turn, reduced levels have been consistently related to weak intercellular adhesion and cell expansion (Knox et al., 1990; Iwai et al., 2002; Majewska-Sawka et al., 2004), as they are much more accessible for polygalacturonases that remodel the pectin matrix (Darley et al., 2001). In embryogenic microspores (Figure 4), we observed very low levels of low and partially methyl-esterified pectins, together with higher levels of high methyl-esterified pectins (recognized by JIM5, LM7 and JIM7, respectively), indicating a low ability for intercellular adhesion. In summary, the specific xyloglucan and pectin profiles of embryogenic microspores produce weak, unstable walls with loose intercellular adhesion and prone to rupture. This was confirmed by their irregular structure and frequent gaps (Figure 3C). All these cell wall features facilitate cell expansion and microspore growth, and are essential for other processes inherent to microspore embryogenesis and DH generation, such as genome doubling through nuclear fusion (Seguí-Simarro and Nuez, 2008; Corral-Martínez et al., 2011; Parra-Vega et al., 2015b).

## Concluding Remarks

During the first stages of the developmental switch, induced microspores undergo profound cell wall changes. In this study, we demonstrated that direct consequences of induction are (1) the compositional remodeling of the preexisting intine walls of microspores, and (2) the formation of unusual walls with a unique structure and composition. The cell walls formed *de novo* in embryogenic microspores (the subintinal layer and inner cell walls) are remarkably similar not only in terms of ultrastructure, but also of AGP, pectin and xyloglucan composition, and different from existing walls (the intine) and walls of MDEs. This adds to our previous observations on callose and cellulose composition (Rivas-Sendra et al., 2019). These special walls, with a unique structure and composition. define their developmental fate at three levels. First, the subintinal layer forms an osmoprotective barrier that preserves viability of embryogenic cells (Rivas-Sendra et al., 2019). Second, the abundance of JIM13-crossreacting AGPs would contribute to determine their competence for embryogenesis, Third, the composition of the existing and newly made walls makes them weak, plastic and prone to rupture, which is a requisite for the well-documented occurrence of genome doubling by nuclear fusion (Parra-Vega et al., 2015b).

## Data Availability

All datasets generated for this study are included in the manuscript and/or the supplementary files.

## Author Contributions

JS-S performed the sample production and processing and manuscript writing. PC-M performed the immunolabeling and TEM observations. AD did the TEM observations and manuscript revision.

## Conflict of Interest Statement

The authors declare that the research was conducted in the absence of any commercial or financial relationships that could be construed as a potential conflict of interest.
